# Association between living arrangements and health risk behaviors among the Hakka older adults in Fujian, China

**DOI:** 10.1186/s12889-023-17107-3

**Published:** 2023-12-01

**Authors:** Xiaojun Liu, Lingling Zhang, Huajing Chang, Mengshi Chen, Yimin Huang

**Affiliations:** 1 Department of Health Management, School of Health Management, Fujian Medical University, 350122 Fuzhou, Fujian China; 2 Department of Epidemiology and Health Statistics, School of Public Health, Fujian Medical University, 350122 Fuzhou, Fujian China; 3 Hunan Provincial Key Laboratory of Clinical Epidemiology, Xiangya School of Public Health, Central South University, 410078 Changsha, Hunan China

**Keywords:** Living arrangements, Health risk behaviors, Chinese Hakka older adults, CHRQLS-OA 2018

## Abstract

**Background:**

Behavioral lifestyles are important social determinants of health. The impact of changes in living arrangements on behavioral lifestyles is currently under-explored. This study aims to examine the association between living arrangements and health risk behaviors among the Hakka older adults.

**Methods:**

Data were extracted from China’s Health-Related Quality of Life Survey for Older Adults 2018. Living arrangements were divided into five categories: living alone, living with spouse only, living with child, mixed habitation, and others. Five health risk behaviors, including unhealthy dietary patterns, drinking, smoking, irregular sleep practices, and physical inactivity were measured. Logistic regression analysis was used to assess the association between living arrangements and specific health risk behaviors, and generalized linear models were established to test the association between living arrangements and the number of health risk behaviors.

**Results:**

A total of 1,262 Hakka older adults were included in this study. Compared to those living alone, those living with spouse only were less likely to have unhealthy dietary patterns (*OR* = 0.45, *P* < 0.05) and drinking (*OR* = 0.50, *P* < 0.05), those living with the child were less likely to experience unhealthy dietary patterns (*OR* = 0.35, *P* < 0.001), drinking (*OR* = 0.32, *P* < 0.001), smoking (*OR* = 0.49, *P* < 0.05), and physical inactivity (*OR* = 0.13, *P* < 0.01). Moreover, those who were living with child (*β* = -0.78, *P* < 0.001) or mixed habitation (*β* = -0.33, *P* < 0.05) tended to engage in fewer health risk behaviors than those living alone.

**Conclusions:**

This study suggests significant differences in health risk behaviors among the Hakka older adults with different living arrangements. Living with the child could reduce the occurrence of health risk behaviors in the Hakka older adults and thus maintain their health status.

## Background

The ageing of the global population has become a major trend in the development of the 21st century. The global population aged 65 and over was 702.9 million in 2019 and which is expected to reach 1.55 billion by 2050 [1]. For a long time to come, China’s ageing population will also be a fundamental national situation. The results of the seventh national census show that the number of people aged over 60 in China reach 264.02 million in 2020, accounting for 18.70% of the whole population, which means that the ageing of China’s population has continued to deepen [2]. Due to rising life expectancy and the one-child policy implemented to curb excessive population growth, China’s declining fertility and mortality rates have the negative consequence of accelerating population ageing [3]. As China’s aging process accelerates, challenges are prevailing, especially the rising health problems among the elderly population. The quality of life of the elderly in China is concerning: only a third of the elderly population is in good health [4]. Population aging is strongly associated with the increased prevalence of chronic disease and disability [5]. The National Health Commission of the People’s Republic of China (PRC) reported that about 190 million order adults in China have chronic diseases, about 40 million are disabled or semi-disabled, and about 15 million have been diagnosed with dementia [6]. In China, which has the largest older population, the issue of unhealthy longevity has become increasingly prominent. Cancer, cardiovascular disease, and diabetes are major challenges faced by the elderly in China and will increase the burden on existing families and public health systems as the population ages [7].

Since behaviors are the key factors that can influence the onset and progression of chronic diseases, behavioral interventions are considered to be the most effective and economic measures for disease control and are widely used to improve the health status of the elderly population. Previous studies have reported that health behaviors have a positive impact on the prevention of chronic diseases such as cancer and diabetes during the ageing process, and that the improvement of appropriate health behaviors is important for the prevention of chronic diseases, especially among older adults [8]. Common behavioral intervention methodologies such as health education initiatives, lifestyle changes and providing social support contribute to the health benefits of healthy ageing [9]. From previously published studies, the number of health risk behaviors was positively associated with the risk of developing disease and death [10–13]. There were also synergistic effects between multiple behaviors [14, 15], such as smoking and alcohol consumption, which have a combined effect on the disease when present together [16, 17]. Moreover, the co-occurrence of multiple health risk behaviors was more pronounced in the elderly population [18]. Additionally, a study showed that a change in one risk behavior is related to a change in another behavior [19]. Therefore, for older adults, the health management targeted on chronic disease related multiple risk behaviors is beneficial and urgently needed.

Previous studies have focused on the factors that influence elderly adults’ health risk behaviors. Some research noted that age, gender, and household income are confirmed as having a significant effect on participating in health risk behaviors [20, 21]. Besides, other studies found that marital status, economic income, and educational attainment are major influencing factors of health risk behaviors [22, 23]. Similarly, there was also a study reported that the living arrangements of seniors are an important determinant of health risk behaviors [24]. The study suggested that living alone means living at risk, people who live with a spouse or children can get family support, which was associated with a lower likelihood of health risk behaviors [24]. However, there were also studies claiming that the management of health risk behaviors of the elderly living alone may be better because of the increasing awareness of self-realization and health responsibility [25]. Especially, Kim et al. [26] found that those living with others may also increase the prevalence of specific health risk behaviors and be reported to make fewer maintenance changes regarding no alcohol consumption compared to those living alone. Thus, it is necessary to recognize the differences in health due to living arrangements and develop new strategies to improve the health of older adults.

In recent decades, under accelerated social development and economic growth, the living arrangements of Chinese older adults have significantly changed [27]. In 1982, nearly 75% of Chinese older adults lived with their children, and by 2011, more than half of Chinese older adults were separated from their children [28]. Moreover, it is worth noting that the proportion of elderly living alone is rapidly increasing in China, reaching over 50% [29]. The empirical evidence indicated that older Chinese people were increasingly living alone or living with spouses only [30]. The dramatic changes in the living arrangements of older adults have given rise to widespread concern in the community about the health of older adults. Living arrangements for older adults are different in different parts of China [31], and being in different regions with different cultures could impact people’s health risk behaviors [32]. The Hakka elderly population, which was selected for this study, had developed their unique customs, culture, and habits in a specific historical and geographical context [33]. The Hakka are a Han ethnic group with distinct traditions and cultures, living mainly in the provinces of Guangdong, Jiangxi and Fujian in China [34]. For historical reasons, they have been forced to migrate south many times, leaving them with limited access to resources [35]. As a result, poor living conditions have forced them to develop unique health-related behaviors to fight disease and maintain health. In addition, the living arrangements of the Hakka people gradually changed from the traditional extended family to private nuclear families. Research on the association between living arrangements and health risk behaviors among the Hakka older adults is important in designing effective health intervention programs to modify the known health risks.

Although the study on living arrangements and health risk behaviors was persuasive [24], limited works have explored how health risk behaviors vary across living arrangements among the Hakka older adults in China. So far, there are as many as 100 million Hakka populations worldwide, yet few studies have specifically targeted this population [36]. It is unclear whether the association between living arrangements and health risk behaviors could be found in the Hakka people. Moreover, a review of the literature suggested that many researchers have focused only on one behavior, while few have addressed multiple behaviors [37], which to some extent, reduces the effectiveness of health interventions and management. Therefore, this study specifically selected five health risk behaviors, specifically including unhealthy dietary patterns, drinking, smoking, irregular sleep practices, and physical inactivity, aims to examine the association between living arrangements and specific health risk behaviors and the number of health risk behaviors, and further to provide strategies for developing health risk behavior control and intervention measures to improve the health status of the Hakka older adults.

## Materials and methods

### Study population

The data used in this paper were derived from China’s Health-Related Quality of Life Survey for Older Adults 2018 (CHRQLS-OA 2018). The CHRQLS-OA 2018 was a cross-sectional survey organized by the Global Health Institute of Wuhan University during the Spring Festival in 2018, aiming to collect data on the status of health-related quality of life among the elderly aged 60 years old and over in China. The survey collected information including participants’ socio-demographic characteristics, health-related quality of life, health-related behaviors and lifestyles, etc. Details of the CHRQLS-OA 2018 are available in previous work [38]. The target population of this study is the Hakka older adults, aiming to examine the association between living arrangements and health risk behaviors among the Hakka older adults. Thus, a sample of 1,262 Hakka older adults aged 60 years and over from Ninghua, Fujian -- commonly known as the cradle of the Hakka were selected from the general database of the CHRQLS-OA 2018. A detailed description of the inclusion and exclusion criteria for participants and the investigation process can be found in our previous publication [39].

### Living arrangements

The CHRQLS-OA 2018 questionnaire enquired about how many people lived in the household and their relationship with the respondents. Thus, five categories of living arrangements of the participants were generated as follows: (1) living alone, which mainly includes elderly adults who were married but separated for various reasons and were living alone at the time of the survey; (2) living with spouse only; (3) living with child, which means the respondents were currently living with one child, regardless of how many children there were. This subgroup also includes older adults who had spouses living together or separated; (4) mixed habitation, which means the elderly respondents were currently living in rotation with multiple children, or the elderly respondents were currently living with more than one child in one house; and (5) other arrangements, including living with grandchildren only, living with others (i.e., siblings and other relatives), and nursing home. Living alone was consistently used as the reference group in data analysis.

### Health risk behaviors

The dependent variables in this study included five health risk behaviors, including unhealthy dietary patterns, drinking, smoking, irregular sleep practices, and physical inactivity. With respect to the CHRQLS-OA 2018 questionnaire, according to the *Health China Action Plan (2019–2030)* [40], health risk behaviors in the present study were defined as follows: (1) unhealthy dietary patterns, i.e., participants self-confessed skipping breakfast or having an unbalanced diet including too much meat intake, insufficient intake of vegetables and fruit, and vegetarian status; (2) drinking, i.e., respondents self-reported drinking more than one time per week, while current non-drinkers were those who had quit drinking or never drank; (3) smoking, i.e., participants self-admitted to smoking at least one cigarette per week, and current non-smokers were those who had quit smoking or never smoked; (4) irregular sleep practices, i.e., individuals self-reported sometimes, rarely, or never sleeping regularly, or that they have irregular times of falling asleep and waking up each day, as well as irregular sleep duration; (5) physical inactivity, i.e., individuals who self-reported doing exercise less than three times a week and less than 30 min per time. We finally calculated the total number of these five health risk behaviors. The number of multiple health risk behaviors ranged from 0 (none of the five selected health risk behaviors occurred) to 5 (all five of the selected health risk behaviors occurred).

### Covariates

For this study, the participants’ socio-demographic characteristics were considered confounders and were used for model adjustments. Socio-demographic characteristics included sex, age, marital status, current residence, educational level, average annual household income (China Yuan, CNY), self-rate health status, and medical insurance. Age was further divided into five groups: 60–64, 65–69, 70–74, 75–79, and over 80. Marital status was classified as married (married/cohabitating) and others (unmarried/widowed/divorced/separated). Current residence was grouped into village, town, and county. Educational level was classified into four groups: illiterate, literacy class/ home school, primary school, and junior high school or above. Average annual household income (CNY) was categorized into five groups: 15,000 and lower, 15,001–30,000, 30,001–45,000, 45,001–60,000, and 60,001 and higher. Self-rate health status was categorized as very good/ good, general, very poor. Medical insurance was classified as urban and rural residents’ basic medical insurance, urban employees basic medical insurance, and uninsured/ unknown.

### Statistical analysis

Data analysis in three steps. Firstly, a descriptive analysis of the socio-demographic characteristics of participants, five selected health risk behaviors, and the number of health risk behaviors were carried out with frequencies and proportions. Secondly, a binary logistic regression model analysis was used to assess the relationship between living arrangements and five specific selected health risk behaviors, and both the crude odds ratios (cOR) and adjusted odds ratios (aOR) with associated 95% confidence intervals (CIs) were calculated. Finally, generalized linear models were used to test the relationship between living arrangements and the number of health risk behaviors, and the coefficient (*β*) with associated 95% CI were presented both in the crude model and adjusted model. The adjusted model was adjusted for confounders that had a significant association with the dependent variables. The Statistical Package for the Social Sciences (SPSS) version 25.0 (SPSS Inc., Chicago, IL, USA) was employed to run all statistical analyses, with a significance level of 0.05.

## Results

### Socio-demographic characteristics of the study participants

A total of 1,262 Hakka older adults, consisting of 613 men (48.57%) and 649 (51.43%) women, were involved in the study. Table 1 demonstrates the demographic data of the participants. The majority of participants (82.01%) were between 60 and 79 years old. Most participants were married or cohabiting (66.80%), currently living in the county (40.41%), and illiterates accounted for more than half of the population. The participants’ family per capita annual incomes ranged from 15,001 to 30,000 yuan (27.66%) and 30,001 to 45,000 yuan (26.86%), accounting for the majority of the sample. There were 56.34% of the subjects self-reported their health status as general, and 78.84% had the medical insurance of urban and rural residents with basic medical insurance. Of the 1,262 participants, around 8.24% of respondents were living alone, 31.70% of respondents were living with a spouse only, 34.47% of respondents were living with the child, 18.62% of respondents were mixed habitation whereas 6.97% of them had other living arrangements.

**Table 1:** Characteristics of the study participants by living arrangements (n, %)

Variables	Living alone	Living with spouse only	Living with child	Mixed habitation	Others	Total
**Sex** (*χ*² = 56.097)***						
Male	34 (5.55)	237 (38.66)	226 (36.87)	93 (15.17)	23 (3.75)	613 (48.57)
Female	70 (10.79)	163 (25.11)	209 (32.20)	142 (21.88)	65 (10.02)	649 (51.43)
**Age (years)** (*χ*² = 192.258)***						
60–64	15 (4.21)	105 (29.50)	164 (46.07)	38 (10.67)	34 (9.55)	356 (28.21)
65–69	18 (7.26)	92 (37.09)	102 (41.13)	27 (10.89)	9 (3.63)	248 (19.65)
70–74	16 (7.05)	114 (50.22)	37 (16.30)	46 (20.26)	14 (6.17)	227 (17.99)
75–79	22 (10.79)	70 (34.31)	50 (24.51)	55 (26.96)	7 (3.43)	204 (16.16)
≥ 80	33 (14.54)	19 (8.37)	82 (36.12)	69 (30.40)	24 (10.57)	227 (17.99)
**Marital status** (*χ*² = 462.947)***						
Married	3 (0.36)	380 (45.08)	332 (39.38)	87 (10.32)	41 (4.86)	843 (66.80)
Others	101 (24.11)	20 (4.77)	103 (24.58)	148 (35.32)	47 (11.22)	419 (33.20)
**Current residence** (*χ*² = 140.477)***						
Village	62 (12.97)	113 (23.64)	122 (25.52)	125 (26.15)	56 (11.72)	478 (37.88)
Town	28 (10.22)	76 (27.74)	97 (35.40)	58 (21.17)	15 (5.47)	274 (21.71)
County	14 (2.75)	211 (41.37)	216 (42.35)	52 (10.20)	17 (3.33)	510 (40.41)
**Educational level** (*χ*² = 378.773)***						
Illiterate	92 (13.65)	123 (18.25)	171 (25.37)	206 (30.56)	82 (12.17)	674 (53.41)
Literacy class/ home school	1 (0.52)	70 (36.46)	93 (48.44)	24 (12.50)	4 (2.08)	192 (15.21)
Primary school	0 (0.00)	84 (43.75)	101 (52.60)	5 (2.61)	2 (1.04)	192 (15.21)
≥ Junior high school	11 (5.39)	123 (60.30)	70 (34.31)	0 (0.00)	0 (0.00)	204 (16.17)
**Average annual household income (CNY)** (*χ*² = 274.313)***						
15,000 and lower	53 (20.39)	60 (23.08)	42 (16.15)	78 (30.00)	27 (10.38)	260 (20.60)
15,001–30,000	51 (14.61)	83 (23.78)	99 (28.37)	89(25.50)	27 (7.74)	349 (27.66)
30,001–45,000	0 (0.00)	112 (33.04)	159 (46.90)	46 (13.57)	22 (6.49)	339 (26.86)
45,001–60,000	0 (0.00)	88 (41.12)	98 (45.79)	19 (8.88)	9 (4.21)	214 (16.96)
60,001 and higher	0 (0.00)	57 (57.00)	37 (37.00)	3 (3.00)	3 (3.00)	100 (7.92)
**Self-rate health status** (*χ*² = 165.076)***						
Very good/ good	12 (3.21)	143 (38.24)	157 (41.98)	37 (9.89)	25 (6.68)	374 (29.64)
General	43 (6.05)	222 (31.22)	249 (35.02)	143 (20.11)	54 (7.60)	711 (56.34)
Very poor	49 (27.68)	35 (19.78)	29 (16.39)	55 (31.07)	9 (5.08)	177 (14.02)
**Medical insurance** (*χ*² = 172.613)***						
URRBMI	96 (9.65)	251 (25.23)	334 (33.57)	230 (23.11)	84 (8.44)	995 (78.84)
UEBMI	2 (1.21)	75 (45.46)	81 (49.09)	5 (3.03)	2 (1.21)	165 (13.08)
Uninsured/ Unknown	6 (5.88)	74 (72.55)	20 (19.61)	0 (0.00)	2 (1.96)	102 (8.08)
**Total**	104 (8.24)	400 (31.70)	435 (34.47)	235 (18.62)	88 (6.97)	1262 (100.00)

Notes: ^*^ = *P* < 0.05, ^**^ = *P* < 0.01, ^***^ = *P* < 0.001. CNY = China Yuan, URRBMI = Urban and rural residents’ basic medical insurance, UEBMI = Urban employees basic medical insurance

### Information on health risk behaviors of participants with different living arrangements

Table 2 shows the characteristics of the participants’ five specific health risk behaviors with different living arrangements. Of those Hakka older adults who lived alone, the most prevalent health risk behavior was physical inactivity (98.08%), followed by irregular sleep practices (83.65%), and then unhealthy diet patterns (75.96%). Among those who lived with their spouse only or with the child, drinking is the most common behavior, accounting for 53.75% and 37.01%, respectively. Of those older adults in mixed habitation, 67.23% had an unhealthy diet, and 80.43% had physical inactivity, while for those in other arrangements, 73.86% were irregular sleep practices, and 72.73% were physical inactivity.

Table 3 shows the characteristics of the number of health risk behaviors in participants with different living arrangements. Among those Hakka older adults who lived alone, 34.62% reported three health risk behaviors, and 27.88% reported five health risk behaviors. Of those who lived with their spouse only, 32.25% had one health risk behavior, and 24.50% had two health risk behaviors. Among those who lived with the child, 32.25% were no health risk behaviors occurred, and 32.25% were one health risk behavior. Of those older adults in mixed habitation, 36.60% had three health risk behaviors, while among those in other living arrangements, 25.00% reported two health risk behaviors.

**Table 2:** Health risk behaviors in participants with different living arrangements (n, %)

Living arrangements	Unhealthy dietary patterns	Drinking	Smoking	Irregular sleep practices	Physical inactivity	Total
Living alone	79 (75.96)	60 (57.69)	36 (34.62)	87 (83.65)	102 (98.08)	104 (8.24)
Living with spouse only	153 (38.25)	215 (53.75)	92 (23.00)	128 (32.00)	150 (37.50)	400 (31.70)
Living with child	138 (31.73)	161 (37.01)	62 (14.25)	134 (30.81)	133 (30.58)	435 (34.47)
Mixed habitation	158 (67.23)	82 (34.89)	60 (25.53)	149 (63.40)	189 (80.43)	235 (18.62)
Others	58 (65.91)	35 (39.77)	32 (36.36)	65 (73.86)	64 (72.73)	88 (6.97)
Total	586 (46.44)	553 (43.82)	282 (22.35)	563 (44.61)	638 (50.55)	1262(100.00)

**Table 3:** The number of health risk behaviors in participants with different living arrangements (n, %)

Living arrangements	0	1	2	3	4	5
Living alone	0 (0.00)	5 (4.81)	15 (14.42)	36 (34.62)	19 (18.27)	29 (27.88)
Living with spouse only	63 (15.75)	129 (32.25)	98 (24.50)	49 (12.25)	39 (9.75)	22 (5.50)
Living with child	133 (30.58)	140 (32.18)	69 (15.86)	47 (10.80)	21 (4.83)	25 (5.75)
Mixed habitation	11 (4.68)	45 (19.15)	36 (15.32)	86 (36.60)	22 (9.36)	35 (14.89)
Others	7 (7.96)	10 (11.36)	22 (25.00)	16 (18.18)	13 (14.77)	20 (22.73)

### Association between specific health risk behaviors and living arrangements

The logistic regression was used to determine the association between a participant’s specific health risk behaviors and living arrangements, as Table 4 shows. In crude and adjusted models, the health risk behaviors were significantly related to different living arrangements. After controlling for other covariates (Socio-demographic information), the Hakka older adults who lived with a spouse only, in contrast to those living alone, were less likely to develop unhealthy dietary patterns (*OR* = 0.45, 95% *CI* = 0.24 to 0.86) and drinking behaviors (*OR* = 0.50, 95% *CI* = 0.27 to 0.92). Moreover, those who lived with the child had lower odds of developing unhealthy dietary patterns (*OR* = 0.35, 95% *CI* = 0.19 to 0.64), drinking (*OR* = 0.32, 95% *CI* = 0.18 to 0.57), smoking (*OR* = 0.49, 95% *CI* = 0.26 to 0.95), and physical inactivity behaviors (*OR* = 0.13, 95% *CI* = 0.03 to 0.58) as compared to those who lived alone. Among those in mixed habitation (*OR* = 0.32, 95% *CI* = 0.19 to 0.55) and in other living arrangements (*OR* = 0.44, 95% *CI* = 0.22 to 0.87) had a significantly lower chance of experiencing drinking behavior as compared to those lived alone.

**Table 4:** Binary logistic regression analysis testing the association between specific health risk behaviors and living arrangements

Crude model	Unhealthy dietary patterns		Drinking		Smoking		Irregular sleep practices		Physical inactivity	
	cOR	95% CI	cOR	95% CI	cOR	95% CI	cOR	95% CI	cOR	95% CI
Living alone	1		1		1		1		1	
Living with spouse only	0.20	(0.12, 0.32)^***^	0.85	(0.55, 1.32)	0.56	(0.35, 0.90)^*^	0.09	(0.05, 0.16)^***^	0.01	(0.00, 0.05)^***^
Living with child	0.15	(0.09, 0.24)^***^	0.43	(0.28, 0.67)^***^	0.31	(0.19, 0.51)^***^	0.09	(0.05, 0.15)^***^	0.01	(0.00, 0.04)^***^
Mixed habitation	0.65	(0.38, 1.10)	0.39	(0.25, 0.63)^***^	0.65	(0.39, 1.07)	0.34	(0.19, 0.61)^***^	0.08	(0.02, 0.34)^***^
Others	0.61	(0.33, 1.15)	0.48	(0.27, 0.86)^*^	1.08	(0.60, 1.95)	0.55	(0.27, 1.12)	0.05	(0.01, 0.23)^***^
**Adjusted model**	**Unhealthy dietary patterns**		**Drinking**		**Smoking**		**Irregular sleep practices**		**Physical inactivity**	
	aOR	95% CI	aOR	95% CI	aOR	95% CI	aOR	95% CI	aOR	95% CI
Living alone	1		1		1		1		1	
Living with spouse only	0.45	(0.24, 0.86)^*^	0.50	(0.27, 0.92)^*^	1.68	(0.84, 3.35)	0.80	(0.38, 1.69)	0.30	(0.06, 1.36)
Living with child	0.35	(0.19, 0.64)^***^	0.32	(0.18, 0.57)^***^	0.49	(0.26, 0.95)^*^	0.53	(0.26, 1.09)	0.13	(0.03, 0.58)^**^
Mixed habitation	0.92	(0.51, 1.65)	0.32	(0.19, 0.55)^***^	0.68	(0.37, 1.24)	0.64	(0.32, 1.28)	0.46	(0.10, 2.11)
Others	0.98	(0.49, 1.98)	0.44	(0.22, 0.87)^*^	1.63	(0.78, 3.41)	1.44	(0.63, 3.32)	0.35	(0.07, 1.69)

Notes: ^*^ = *P* < 0.05, ^**^ = *P* < 0.01, ^***^ = *P* < 0.001. Adjusted model adjusted for sex, age, marital status, current residence, educational level, average annual household income (China Yuan, CNY), self-rate health status, and medical insurance

### Association between the number of health risk behaviors and living arrangements

Generalized linear regression analyses were presented to test if the number of health risk behaviors was associated with different living arrangements (Table 5). Overall, as compared with the reference group (living alone), individuals living with the child (*β* = -0.78, 95% CI = -1.07 to -0.48) and mixed habitation (*β* = -0.33, 95% CI = -0.61 to -0.05) exhibited fewer health risk behaviors in the adjusted model. Notably, from the result of the test between Hakka older adults living alone and living with a spouse only, the *P*-value is less than 0.05, but close to 0.05. Therefore, it is difficult to conclude that there is no significant difference in multiple health risk behaviors between older adults who live alone and those who live with their spouses only.

**Table 5:** Generalized linear models testing the association between the number of health risk behaviors and living arrangements

Living arrangements	Crude model, *β* (95% CI)	Adjusted model, *β* (95% CI)
Living alone	1.00 (Reference)	1.00 (Reference)
Living with spouse only	-1.66 (-1.96, -1.35)^***^	-0.30 (-0.62, 0.01)
Living with child	-2.06 (-2.36, -1.76)^***^	-0.78 (-1.07, -0.48)^***^
Mixed habitation	-0.79 (-1.11, -0.46)^***^	-0.33 (-0.61, -0.05)^*^
Others	-0.61 (-1.01, -0.22)^**^	-0.01 (-0.35, 0.34)

Notes: ^*^ = *P* < 0.05, ^**^ = *P* < 0.01, ^***^ = *P* < 0.001. Adjusted model adjusted for sex, age, marital status, current residence, educational level, average annual household income (China Yuan, CNY), self-rate health status, and medical insurance

## Discussion

With the acceleration process of modernization, the living arrangements of older adults are changing, and the proportion of older adults living alone is increasing [41]. The living arrangements of older adults delegate a significant component of the social environment and have been theorized to impact health through their affect health behaviors [42]. Results from a representative sample of Hakka older adults indicate that differences in health risk behaviors exist across the five living arrangement categories tested in this study. This study found that compared with both living with a spouse only and living with the child, Hakka older adults who living alone were more likely to have unhealthy dietary patterns and drinking. Similarly, Hakka older adults who living alone exhibited more health risk behaviors compared to those living with the child and mixed habitation. Therefore, this means that individuals who lived alone had the highest odds of health risk behaviors, which is consistent with prior studies [24, 26]. Although recent research has conveyed the active thoughts that many older adults have about living alone, this is for older people who can take care of themselves with sufficient resources and economic conditions [43, 44]. The possible reason for this is that the association between living alone and health behavior may indicate a selection effect: Individuals who are able to live independently have fewer health risk behaviors that allow them to do so. However, if older people are forced to live alone but lack adequate financial conditions and family and social support, living alone can have negative consequences. While the Hakka older adults in Fujian who were included in this study mainly lived in underdeveloped areas, and the impact of living alone is negative. Thus, the vulnerable group of Hakka older adults living alone who are at risk of participating in health risk behaviors should be more noticed. It is necessary for the family, community, and governments to provide combined actions and support to improve their health and well-being.

It is generally accepted that living with family is better than living alone in constraining health risk behaviors of older adults due to the benefits of shared family support and both direct and indirect social control [24, 30]. Living with family usually means living with a spouse and children. Our results found that Hakka older adults both living with a spouse only and living with the child are less likely to have unhealthy dietary patterns compared to living alone. This is similar to a previous study [45], which found that the unhealthy dietary pattern was more frequent in individuals living alone than in those living with others. The possible reason is that if the Hakka older adults eat with their spouse or a single family, they can consume more food types and ensure regular meals. Moreover, it has been shown that eating alone can negatively affect eating patterns and diet quality and may lead to undesirable dietary behaviors [46]. Furthermore, Hakka people live in the mountains and have limited transport, resulting in a single source of food and less food consumption, which makes them more likely to engage in unhealthy dietary patterns if they are eating alone. In addition, we also found that the Hakka older adults living alone showed the highest rate of drinking behavior, which is consistent with the study by Zhang et al. [24]. A previous study showed that humans living alone suffered a higher likelihood of feeling lonely than those living with someone else [47]. Furthermore, people engage in alcohol consumption often to cope with feelings of isolation and unworthiness [47, 48]. Previous research has shown that the unique socio-ecological environment of the Hakka region has shaped the wine-making culture, allowing them to drink more and thus reduce their loneliness [34]. Thus, it is easy to understand that the Hakka older adults avoiding living alone helps to limit drinking behavior.

Besides, we also found that living with the child was associated with a significantly lower risk of smoking behavior and physical inactivity for the Hakka older adults, while this benefit was not observed in living with a spouse and in mixed habitation. However, the findings from Zhang et al. [24] have shown inconsistent evidence that the likelihood of older adults smoking was lower for older men living with the children and for women living with a spouse only or with both a spouse and children. Similarly, a study indicates that older adults living with others (including spouses, children, and others) were more likely to enjoy more leisure-time physical activity than those living alone [49]. One possible explanation for the differences in these findings could be due to the impact of different regions with different cultures. Another possible explanation is that children help their parents to avoid smoking and physical inactivity, and to develop better health behaviors. In addition, our finding that irregular sleep practices did not differ by living arrangements and spouses and children could not limit irregular sleep behavior among the Hakka older adults contradicts the previous study [50]. Many studies reported that the social isolation experienced by older adults who lived alone had a higher negative influence on sleep quality compared with those living with others [51, 52]. Therefore, the possible reason is that compared with the negative effects of living alone, factors such as age, economic level, and sleep environment have a substantial impact on the sleep quality of the Hakka older adults [50]. Thus, it’s imperative to conduct further research to investigate whether the irregular sleep practices of older adults are related to living arrangements.

Chinese people respect filial piety and they believe that children are supposed to take care of their parents and provide them with financial and emotional support [53–55]. The Hakka people have survived in isolated mountain conditions for a long time, usually in closed “castled houses” (tu lou) [35, 56], which allows for more intimate interaction between parents and children. In particular, this study classifies the living arrangements into living with the child and living with multiple children, which brings an interesting finding. Those living with the child are more effective to limit health risk behaviors than mixed habitation, both for specific health risk behaviors and for protection against multiple numbers of behaviors. This situation is contrary to the proverb of “more children, more happiness” that the Chinese always advocate. On the one hand, a possible reason is the impact of intergenerational ambivalence. The intergenerational support means that children can provide health information to older people and urge them to engage in healthy behaviors as a way to improve their health status [57]. While intergenerational ambivalence believes that older people and their children have different philosophies and that living together can bring about some conflict, leading to restrictions on older people’s behavioral lifestyles and poorer health [58]. For the Hakka older adults, mixed habitation means that conflicts with children are more likely to occur, which has adverse effects. On the other hand, children with more siblings are less likely to provide support to their parents than singleton children, which has been confirmed in the study by Guo et al. [59].

Compared to having one single health-risk behavior, engaging in multiple health-risk behaviors were linked with increased morbidity and mortality of chronic diseases [60, 61]. Additionally, mounting evidence shows that changes in one risk behavior are related to changes in another behavior [62]. Consequently, there is a potential benefit in multiple health risk behavior interventions [63]. It is worth noting that in this study, we observed that the Hakka older adults living with the child and those living in mixed habitation exhibited fewer health risk behaviors compared to those living alone. For one thing, children can help parents reduce health risk behaviors by taking actions such as disseminating health information, persuading, and encouraging their older parents not to engage in health risk behaviors [24]. For another thing, as previously described that people who smoked were more likely to be physically inactive and develop an unhealthy diet [37]. In our study, living with the child can limit smoking behavior, which may help prevent the occurrence of multiple health risk behaviors. It is therefore crucial for the Hakka older adults to choose to live with children to change health risk behaviors. Furthermore, a comprehensive package should be designed and implemented in a targeted manner, especially those living alone, combining several related intervention programmes across the full range of health risk behaviors to achieve substantially greater health benefits as a convenient and cost-effective alternative. For example, regular health education on smoking and drinking is conducted to encourage Hakka older adults to avoid smoking and drinking behaviors, as well as encouraging them to develop good eating and sleeping habits, and also regular physical activity. Overall, preventing the occurrence of multiple risk health behaviors will have a significant impact on public health. Moreover, better health behaviors depend on increased health literacy and health knowledge [64]. Nevertheless, the Hakka older adults in this survey lived in the underdeveloped areas, with limited health literacy and less disseminated local health knowledge. Therefore, the Hakka older adults need to promote their health literacy and to develop healthy behaviors and lifestyles to improve their health status.

There are some limitations in this study. Firstly, the data for the study comes from the Hakka population in Ninghua County, Fujian Province, which has its unique lifestyle and habits and is also located in a relatively underdeveloped area. Thus, although this study can provide some policy insights, possible affect the results applicability in other provinces and the country. Secondly, causal effects between the variables and the outcome cannot be ascertained because of its cross-sectional design. Thirdly, the data in this study relied on a self-reported survey format, which inevitably suffers from recall bias. Fourthly, there were limitations to the sleep assessment, which did not use professional sleep scales and did not take into account participants’ sleep quality. Finally, although some variables were controlled in this study, other potential factors could bias the results, such as occupations before retirement, personal savings, and living arrangement preferences, etc.

## Conclusion

To some extent, this paper helps to understand the special group of Hakka older adults, and to explore improvement measures that are different from the general elderly. This study has clearly shown that the different living arrangements of the Hakka older adults are associated with their health risk behaviors. Hakka older adults who lived alone had the highest odds of health risk behaviors. Moreover, living with the child could reduce the occurrence of health risk behaviors in the Hakka older adults and thus maintain their health status. Therefore, customized interventions and programs according to personal living arrangements, as well as scientifically and effectively approach, is needed to promote health behaviors among the Hakka older adults.

## Data Availability

The datasets used and/or analyzed during the current study are available from the first and/ or corresponding author(s) on reasonable request because the datasets contain sensitive material that can identify participant information.

## Abbreviations

OR: Odds ratios
cOR: Crude odds ratios
aOR: Adjusted odds ratios
CHRQLS-OA 2018: China’s Health-Related Quality of Life Survey for Older Adults 2018
CNY: China Yuan
CI: Confidence interval
SPSS: Statistical Package for the Social Sciences
URRBMI: Urban and rural residents’ basic medical insurance
UEBMI: Urban employees basic medical insurance
*β*: coefficient
n: Subsample

## References

[1] Fu J, Huang C, Li S, Xia Y (2023). Evolutionary game analysis of rural public-private partnership older adult care project in the context of population aging in China. Front Public Health.

[2] National Bureau of Statistics. The main data of the seventh national census. [accessed on 12 May 2021]; Available online: http://www.stats.gov.cn/sj/sjjd/202302/t20230202_1896484.html.

[3] Zeng Y, Hesketh T (2016). The effects of China’s universal two-child policy. Lancet.

[4] Zhuori N, Cai Y, Yan Y, Cui Y, Zhao M (2019). Does social support sffect the health ofthe elderly in rural China? A meta-analysis approach. Int J Environ Res Public Health.

[5] Chatterji S, Byles J, Cutler D, Seeman T, Verdes E (2015). Health, functioning, and disability in older adults—present status and future implications. Lancet.

[6] Sun J, Wang J, Li H (2023). Are adverse childhood experiences associated with trajectories of healthy aging? Evidence from China. SSM Popul Health.

[7] Fang EF, Xie C, Schenkel JA, Wu C, Long Q, Cui H (2020). A research agenda for ageing in China in the 21st century (2nd edition): focusing on basic and translational research, long-term care, policy and social networks. Ageing Res Rev.

[8] Isozaki A, Tadaka E (2021). Development of a health behavior scale for older adults living alone receiving public assistance. BMC Public Health.

[9] Chen X, Giles J, Yao Y, Yip W, Meng Q, Berkman L (2022). The path to healthy ageing in China: a Peking University-Lancet Commission. Lancet.

[10] Loef M, Walach H (2012). The combined effects of healthy lifestyle behaviors on all cause mortality: a systematic review and meta analysis. Prev Med.

[11] Johari SM, Shahar S (2014). Metabolic syndrome: the association of obesity and unhealthy lifestyle among Malaysian elderly people. Arch Gerontol Geriatr.

[12] Wang X, Yang X, Li J, Liu F, Chen J, Liu X et al. Impact of healthy lifestyles on cancer risk in the Chinese population. Cancer. 2019;125(12):2099–2106. doi: 0.1002/cncr.31971. 10.1002/cncr.31971 30748010

[13] Hu Z, Qin L, Kaminga AC, Xu H (2020). Relationship between multiple lifestyle behaviors and health-related quality of life among elderly individuals with prediabetes in rural communities in China: a STROBE-compliant article. Med (Baltim).

[14] Liu M, Zhang C, Cai H, Liu F, Liu Y, Li J (2016). The willingness to change risky health behaviors among Chinese rural residents: what we learned from a population-based Esophageal cancer cohort study. PLoS ONE.

[15] Ding D, Rogers K, van der Ploeg H, Stamatakis E, Bauman AE (2015). Traditional and emerging lifestyle risk behaviors and all-cause mortality in middle-aged and older adults: evidence from a large population-based Australian cohort. PLoS Med.

[16] Zhang Z, Ma L, Geng H, Bian Y (2021). Effects of Smoking, and drinking on serum gamma-glutamyl transferase levels using physical examination data: a cross-sectional study in Northwest China. Int J Gen Med.

[17] Hong Z, Pan L, Ma Z, Zhu Y, Hong Z (2019). Combined effects of cigarette Smoking, alcohol drinking and eNOS Glu298Asp polymorphism on blood pressure in Chinese male hypertensive subjects. Tob Induc Dis.

[18] Li Y, Zhang M, Jiang Y, Wu F (2012). Co-variations and clustering of chronic Disease behavioral risk factors in China: China chronic Disease and risk factor surveillance. PLoS ONE.

[19] Ramo DE, Thrul J, Vogel EA, Delucchi K, Prochaska JJ (2020). Multiple health risk behaviors in young adult smokers: stages of change and stability over time. Ann Behav Med.

[20] Walker SN, Volkan K, Sechrist KR, Pender NJ (1988). Health-promoting life styles of older adults: comparisons with young and middle-aged adults, correlates and patterns. ANS Adv Nurs Sci.

[21] Strawbridge WJ, Camacho TC, Cohen RD, Kaplan GA (1993). Gender differences in factors associated with change in physical functioning in old age: a 6-year longitudinal study. Gerontologist.

[22] Moura LR, Torres LM, Cadete MMM, Cunha CF (2018). Factors associated with health risk behaviors among Brazilian adolescents: an integrative review. Rev Esc Enferm USP.

[23] Telias A, Dougan MM, Pignotti GAP (2022). Impact of COVID-19 on health risk behaviors in northern California: a cross-sectional survey. Prev Med Rep.

[24] Zhang J, Wu L (2015). Cigarette Smoking and alcohol consumption among Chinese older adults: do living arrangements matter?. Int J Environ Res Public Health.

[25] Zhang C, Zhu R, Lu J, Xue Y, Hou L, Li M (2018). Health promoting lifestyles and influencing factors among empty nesters and non-empty nesters in Taiyuan, China: a cross-sectional study. Health Qual Life Outcomes.

[26] Kim N, Kim H, Kwon S (2020). Factors associated with different numbers of health behaviors by living arrangements. BMC Public Health.

[27] Huang X, Liu J, Bo A (2020). Living arrangements and quality of life among older adults in China: does social cohesion matter?. Aging Ment Health.

[28] Tang D, Lin Z, Chen F (2020). Moving beyond living arrangements: the role of family and friendship ties in promoting mental health for urban and rural older adults in China. Aging Ment Health.

[29] Zhai Y, Yi H, Shen W, Xiao Y, Fan H, He F (2015). Association of empty nest with depressive symptom in a Chinese elderly population: a cross-sectional study. J Affect Disord.

[30] Zhou Z, Mao F, Ma J, Hao S, Qian ZM, Elder K (2018). A Longitudinal Analysis of the Association between Living Arrangements and Health among older adults in China. Res Aging.

[31] Silverstein M, Cong Z, Li S (2006). Intergenerational transfers and living arrangements of older people in rural China: consequences for psychological well-being. J Gerontol B Psychol Sci Soc Sci.

[32] Morris LJ, D’Este C, Sargent-Cox K, Anstey KJ (2016). Concurrent lifestyle risk factors: clusters and determinants in an Australian sample. Prev Med.

[33] Zhao P, Hou J, Wu H, Zhong M (2018). Analysis of genetic polymorphism of methylenetetrahydrofolate reductase in a large ethnic Hakka population in southern China. Med (Baltim).

[34] Chang H, Ruan W, Chen Y, Cai L, Liu X (2023). Gender differences in the relationship between loneliness and health-related behavioral risk factors among the Hakka elderly in Fujian, China. Front Psychiatry.

[35] Au DT, Wu J, Jiang Z, Chen H, Lu G, Zhao Z (2008). Ethnobotanical study of medicinal plants used by Hakka in Guangdong, China. J Ethnopharmacol.

[36] Xiao Z, Gong R, Chen X, Xiao D, Luo S, Ji Y (2021). Association between serum lactate dehydrogenase and 60-day mortality in Chinese Hakka patients with acute Myeloid Leukemia: a cohort study. J Clin Lab Anal.

[37] Li HCW, Ho LLK, Chung OKJ, Cheung AT, Xia W, Song P (2022). A descriptive study on multiple health-risk behaviors among Chinese adults in Hong Kong. Int J Environ Res Public Health.

[38] Liu X, Chen J, Zhou J, Liu J, Lertpitakpong C, Tan A (2019). The relationship between the number of daily health-related behavioral risk factors and sleep health of the elderly in China. Int J Environ Res Public Health.

[39] Liu X, Liu F, Ruan W, Chen Y, Qu S, Wang W (2022). Mental health status and associated contributing factors among the Hakka elderly in Fujian, China. Front Public Health.

[40] National Health Commission of the People’s Republic of China Government of China,Beijing. : Healthy China Action Plan (2019–2030) [accessed on 6 November 2019]; Available online: http://www.gov.cn/xinwen/2019-07/15/content_5409694.htm.

[41] Wang D, Zheng J, Kurosawa M, Inaba Y, Kato N (2009). Changes in activities of daily living (ADL) among elderly Chinese by marital status, living arrangement, and availability of healthcare over a 3-year period. Environ Health Prev Med.

[42] Russell D (2009). Living arrangements, social integration, and loneliness in later life: the case of physical disability. J Health Soc Behav.

[43] Henning-Smith C, Shippee T, Capistrant B (2018). Later-life disability in environmental context: why living arrangements matter. Gerontologist.

[44] Henning-Smith C, Gonzales G (2020). The relationship between living alone and self-rated health varies by age: evidence from the national health interview survey. J Appl Gerontol.

[45] Kim N, Kim GU, Kim H (2020). Comparative study of dietary patterns by living arrangements: the Korea national health and nutrition examination survey (KNHANES) 2013–2015. Int J Environ Res Public Health.

[46] Kobayashi S, Asakura K, Suga H, Sasaki S, Three-generation Study of Women on Diets and Health Study Group (2017). Living status and frequency of eating out-of-home foods in relation to nutritional adequacy in 4,017 Japanese female dietetic students aged 18–20 years: a multicenter cross-sectional study. J Epidemiol.

[47] Stickley A, Koyanagi A, Roberts B, Richardson E, Abbott P, Tumanov S (2013). Loneliness: its correlates and association with health behaviours and outcomes in nine countries of the former Soviet Union. PLoS ONE.

[48] Bragard E, Giorgi S, Juneau P, Curtis BL (2022). Loneliness and daily alcohol consumption during the COVID-19 pandemic. Alcohol Alcohol.

[49] Yu CY, Hou SI, Miller J (2018). Health for older adults: the role of social capital and leisure-time physical activity by living arrangements. J Phys Act Health.

[50] Chu HS, Oh J, Lee K (2022). The relationship between living arrangements and sleep quality in older adults: gender differences. Int J Environ Res Public Health.

[51] Yu B, Steptoe A, Niu K, Ku PW, Chen LJ (2018). Prospective associations of social isolation and loneliness with poor sleep quality in older adults. Qual Life Res.

[52] Griffin SC, Williams AB, Ravyts SG, Mladen SN, Rybarczyk BD (2020). Loneliness and sleep: a systematic review and meta-analysis. Health Psychol Open.

[53] Cheng ST, Fung HH, Chan AC (2008). Living status and psychological well-being: social comparison as a moderator in later life. Aging Ment Health.

[54] Chen F (2005). Residential patterns of parents and their married children in contemporary China: a life course approach. Popul Res Policy Rev.

[55] Li LW, Zhang J, Liang J (2009). Health among the oldest-old in China: which living arrangements make a difference?. Soc Sci Med.

[56] Luo B, Li F, Ahmed S, Long C (2019). Diversity and use of medicinal plants for soup making in traditional diets of the Hakka in West Fujian, China. J Ethnobiol Ethnomed.

[57] Quashie NT, Andrade FCD, Meltzer G, García C (2022). Living arrangements and intergenerational support in Puerto Rico: are fathers disadvantaged?. J Gerontol B Psychol Sci Soc Sci.

[58] Hogerbrugge MJ, Komter AE (2012). Solidarity and ambivalence: comparing two perspectives on intergenerational relations using longitudinal panel data. J Gerontol B Psychol Sci Soc Sci.

[59] Guo M, Chi I, Silverstein M (2016). Trajectories and determinants of elder care in rural China during an 8-Year period: why having sons makes a difference. Res Aging.

[60] Geller K, Lippke S, Nigg CR (2017). Future directions of multiple behavior change research. J Behav Med.

[61] Noble N, Paul C, Turon H, Oldmeadow C (2015). Which modifiable health risk behaviours are related? A systematic review of the clustering of Smoking, nutrition, alcohol and physical activity (‘SNAP’) health risk factors. Prev Med.

[62] Johnson SK, von Sternberg K, Velasquez MM (2018). Changing multiple health risk behaviors in CHOICES. Prev Med Rep.

[63] Thomas K, Nilsson E, Festin K, Henriksson P, Lowén M, Löf M (2020). Associations of psychosocial factors with multiple health behaviors: a population-based study of middle-aged men and women. Int J Environ Res Public Health.

[64] Liu YB, Liu L, Li YF, Chen YL (2015). Relationship between health literacy, health-related behaviors and health status: a survey of elderly Chinese. Int J Environ Res Public Health.

